# The Role of Krüppel-like Factor 4 in Renal Fibrosis

**DOI:** 10.3389/fphys.2015.00327

**Published:** 2015-11-12

**Authors:** Ben Ke, Afei Zhang, Xianfeng Wu, Xiangdong Fang

**Affiliations:** Department of Nephrology, Nanchang University School of Medicine, Second Affiliated Hospital to Nanchang UniversityNanchang, China

**Keywords:** KLF4, renal fibrosis, inflammation, TGF-β, vascular disease

## Abstract

Chronic kidney disease (CKD) caused by renal fibrosis is an important public health concern. It is therefore necessary to understand the molecular pathogenesis of renal fibrosis in order to develop novel therapeutic strategies. KLF4 is the most extensively studied factor among the various members of the Krüppel-like factor (KLF) family of zinc finger-containing transcription factors. Many studies have demonstrated that KLF4 inhibits the activation of myofibroblasts and exerts an inhibitory effect on fibrosis. However, other studies have indicated that KLF4 may promote renal fibrosis. These controversial results suggest that KLF4 may be crucially involved in the development of renal fibrosis, although the underlying mechanism(s) remain unclear. Here, we summarize the recent progress made in understanding the role of KLF4 in renal fibrosis. Together, these findings suggest that KLF4 may participate in the development of renal fibrosis, but that its inhibition of fibrosis is greater than its promotion of the condition, which suggests that KLF4 may serve as a novel therapeutic target for renal fibrosis.

## Introduction

Renal fibrosis is characterized by excessive proliferation of fibroblasts and increased deposition of extracellular matrix (ECM), which together lead to extensive scarring (fibrotic tubular and glomerular sclerosis), renal artery stenosis and chronic inflammatory cell infiltration (Li et al., 2015). The development of renal fibrosis is a complex process that involves the activation of molecules both intrinsic to the kidney and the infiltrated cells, which results in the deposition of ECM, ultimately leading to the loss of renal function. Tubulointerstitial fibrosis represents the end stage of renal fibrosis.

The Krüppel-like factor (KLF) family consists of 17 zinc finger-containing transcription factors, among which KLF4 is the most extensively studied (Black et al., 2001). KLF4 is essential for development and regulates a variety of processes, such as cell proliferation and differentiation (Black et al., 2001). Many studies have reported that KLF4 inhibits the activation of myofibroblasts and exhibits an inhibitory effect on fibrosis (Yang et al., 2013; Gras et al., 2015). However, in animal models of cardiac hypertrophy, KLF4 promoted myocardial fibrosis (Liao et al., 2010; Zhang et al., 2013). The function of KLF4 in the kidney is still not well understood. In this review, we highlight recent findings on how KLF4 regulates renal fibrosis, with the aim of evaluating the potential of KLF4 as a novel therapeutic target for renal fibrosis.

## The biological characteristics of KLF4

KLF4 was first described by Shields et al. (1996) after identification of the gene from a mouse NIH3T3 cell cDNA library. KLF4 was formerly known as gut-enriched KLF, or epithelial zinc finger KLF, and contains several functional domains, including an N-terminal transcription activation domain that interacts with other proteins, a C-terminal zinc finger structure that combines with the DNA binding domain, and a transcription inhibition zone close to the N-terminal zinc finger structure (Bieker, 2001). KLF4 is expressed in many tissues, tumors, vascular smooth muscle cells (VSMC), and monocytes/macrophages, but it has been reported to be most highly expressed in colon cancer, the lungs (Yoshida and Hayashi, 2014), and the kidneys (Hayashi et al., 2014).

After binding to its specific protein partners, KLF4 activates or inhibits the transcription of target genes. KLF4 thus regulates numerous cellular processes, including proliferation, differentiation, apoptosis, migration, and invasion (Wang et al., 2015). Furthermore, KLF4 plays a specific role in cell reprogramming and differentiation, transforming somatic cells into induced pluripotent stem cells (IPS; D'Anselmi et al., 2013; Yamaguchi et al., 2014).

KLF4 is abundantly expressed in kidney podocyte cells, and its expression is decreased in proteinuric states (Hayashi et al., 2014). In the diabetic kidney, the KLF4 mRNA level is remarkably reduced. In an animal model of diabetic nephropathy, KLF4 expression was significantly decreased in renal tubular cells (Mreich et al., 2015). Renal fibrosis is considered to be one of the major pathological changes that occur during the course of diabetic nephropathy (Arora and Singh, 2013). Together, these findings suggest that a change in the KLF4 level may be closely related to renal fibrosis.

## KLF4 and renal fibrosis

### KLF4 and kidney inflammation

Inflammation is integral to the body's defense, although excessive inflammation is often considered to be the main driving force of fibrosis (Li et al., 2015). Inflammatory cells release large amounts of chemokines and vasoactive factors, such as monocyte chemotactic protein-1 (MCP-1) and angiotensin II, which contribute to the production of pro-fibrotic cytokines after kidney injury (Chung and Lan, 2011). At the site of injury, profibrogenic factors, such as interleukin (IL)-1, MCP-1, and macrophage migration inhibitory factor (MIF), stimulate the generation of myofibroblasts and the deposition of ECM, eventually leading to renal dysfunction (Mack and Yanagita, 2015). Therefore, kidney damage due to persistent inflammation is the originating factor for renal fibrosis (Meng et al., 2014).

KLF4 is involved in renal fibrosis by regulating inflammation (Mreich et al., 2015). MIF and MCP-1 are important inflammatory cytokines associated with kidney disease (Qi et al., 2006; Lan, 2008). It was recently demonstrated that KLF4 reduced inflammation by abrogating the transforming growth factor-β1 (TGF-β1)-induced production of MIF and MCP-1 in human renal tubular cells (Mreich et al., 2015). Consistently, in endothelial cells, KLF4 has shown anti-inflammatory effects by increasing the expression of endothelial nitric oxide synthase, decreasing inflammatory cell adhesion to the endothelial surface and prolonging the clotting time under inflammatory states (Hamik et al., 2007). In macrophages, however, the expression of TGF-β was shown to be regulated by the ratio of the M1 and M2 subtypes of macrophages (López-García et al., 2015), and KLF4 could activate epithelial factors and mediate proinflammatory signals, thereby exhibiting pro-inflammatory activity (Feinberg et al., 2005). The mechanism promoting such inflammation may be related to differentially regulated expression of KLF4 between the two subtypes of macrophages (M1/M2; Liao et al., 2011).

Nuclear factor-kappa B (NF-κB) is an important participant in a broad spectrum of inflammatory networks that regulate the cytokine activity in renal fibrosis (Wu et al., 2015). Upon NF-κB activation, KLF4 binds to the promoters of inflammatory cytokines, such as TNF-α and IL-6, to increase their transcription (Kaushik et al., 2010). In addition, KLF4 interacts with the p65 subunit of NF-κB to activate the transcription of proinflammatory genes and trigger inflammation (Autieri, 2008). KLF4 can also bind to high mobility group box-1 protein, an important mediator of systemic and local inflammatory responses, to achieve pro-inflammatory effects (Liu et al., 2008).

**Figure 1:**
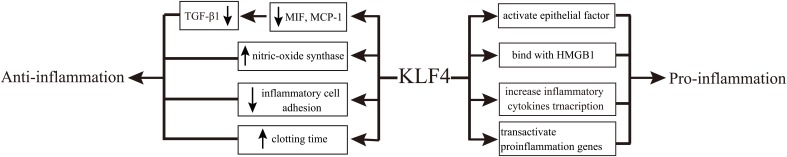
**The role of KLF4 in kidney inflammation**.

However, it remains unclear what function KLF4 plays at the onset of kidney inflammation. In particular, KLF4 generally shows anti-inflammatory effects in epithelial and endothelial cells, while it exhibits pro-inflammatory effects in other cell types (Figure 1).

### KLF4 and TGF-β

The TGF-β superfamily consists of three subtypes: TGF-β1, TGF-β2, and TGF-β3 (Piek et al., 1999). TGF-β plays important roles to promote cell proliferation and differentiation and induce the synthesis of the ECM. It is also known that TGF-β is a major cytokine/growth factor involved in renal fibrosis (Meng et al., 2012). Smad proteins, highly conserved transcription factors, are central to the signal transduction pathways that mediate the numerous effects of the TGF-β superfamily (Massagué, 2012). TGF-β1 strongly stimulates the renal tubular epithelial-to-mesenchymal transition (EMT), which is a crucial process in the development of tubulointerstitial fibrosis (Carew et al., 2012; Xiao et al., 2015).

**Figure 2:**
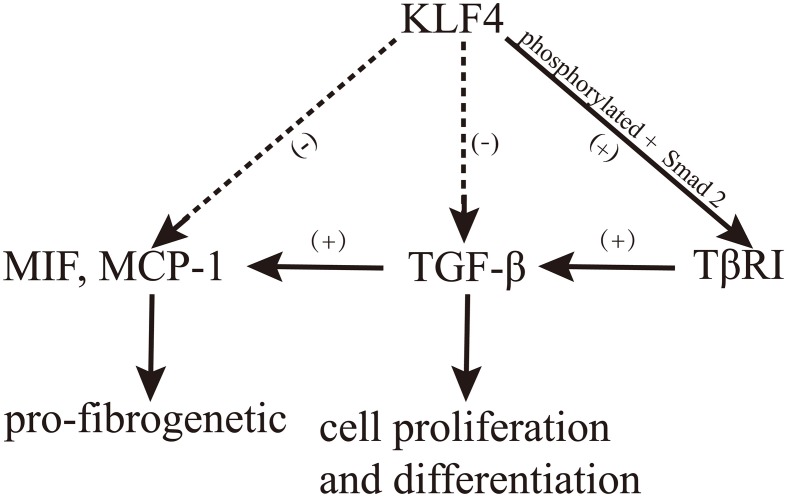
**The role of KLF4 in TGF-β signaling**.

A complex relationship exists between KLF4 and TGF-β, which plays an important role in the development of renal fibrosis. It was recently indicated that the regulation of cell proliferation and differentiation through TGF-β is mediated by KLF4, which binds Smad3 to modulate TGF-β-induced gene expression (Hu et al., 2007). Moreover, the overexpression of KLF4 significantly reduced the production of MIF and MCP-1, which are important mediators of TGF-β-induced renal fibrosis (Mreich et al., 2015). Surprisingly, in cardiac fibroblasts, KLF4 binds with TGF-β1 to upregulate the expression of TGF-β1, while this binding is suppressed by TGF-β signaling in macrophages (King et al., 2003; Figure 2).

TGF-β impacts KLF4 both directly and indirectly. In an indirect manner, TGF-β can quickly reduce the expression of KLF4 by inducing miRNA-143 and miRNA-145 (Davis-Dusenbery et al., 2011). In a direct manner, TGF-β1 induces KLF4 phosphorylation via the Smad and p38 MAPK signaling pathways, and phosphorylated KLF4 can, in turn, interact with Smad2 to cooperatively activate the promoter of TGF-β type I receptor (TβRI; Hu and Wan, 2011). Moreover, Cdh1-anaphase promoting complex (Cdh1/APC), a well-known regulator of mitosis (Peters, 2002), acts as a putative E3 ligase to control TGF-β-induced KLF4 degradation. Both depletion of Cdh1 by RNA interference and stabilization of KLF4 by disruption of its destruction box significantly attenuated the TGF-β-induced ubiquitylation and degradation of KLF4. Additionally, depletion of Cdh1 or stabilization of KLF4 antagonized the TGF-β-induced activation of target gene transcription (Hu and Wan, 2011). Meanwhile, KLF4 is degraded in response to TGF-β signaling through Cdh1/APC, which is catalyzed by KLF4 ubiquitylation (Hu and Wan, 2011). Taken together, these findings indicate that TGF-β plays an instrumental role in inducing KLF4 degradation (Figure 2). On the other hand, the overexpression of KLF4 inhibits the pro-fibrotic effects of TGF-β1 (Mreich et al., 2015).

EMT has been shown to play an active role in renal tubular fibrosis (Galichon and Hertig, 2011). DNA methylation, a type of epigenetic modification in mammals, plays a crucial role in the regulation of gene transcription, which is closely related to EMT (Carmona et al., 2014). DNA methyltransferase 1 (DNMT1) is responsible for the maintenance of pre-existing DNA methylation patterns after replication (Goll and Bestor, 2005). In *in vivo* and *in vitro* models of renal EMT, the KLF4 mRNA and protein levels were reduced compared with the control, and the downregulation of KLF4 was found to be due to DNMT1-mediated KLF4 promoter hypermethylation, which contributed to the progression of EMT in renal epithelial cells (Xiao et al., 2015). In tumor cells, KLF4 also demonstrated the ability to regulate EMT (Chen et al., 2014). In particular, KLF4 activated the transcription of the epithelial cell marker E-cadherin and repressed the expression of a mesenchymal cell marker, snail 2 (slug), by binding to their respective promoters.

### KLF4 and vascular damage

In renal fibrosis, the significant changes in the structure and function of peritubular capillaries and the apoptosis of peritubular capillary endothelial cells give rise to capillary loss, tissue hypoxia and oxidative stress. At the same time, the loss of capillaries causes hypoxia or a reduction in the local nutrient supply, aggravating the renal fibrosis and resulting in kidney damage (Cao et al., 2012; Chapal et al., 2013). Moreover, changes in the renal tubular capillary structure and function lead to renal interstitial inflammation and a decrease or loss of renal tubular capillaries, eventually leading to further renal fibrosis. Thus, vascular damage is an essential factor contributing to renal fibrosis (Yamaguchi et al., 2012).

Great efforts have been made to understand the role of KLF4 in VSMC, and KLF4 is known to inhibit VSMC differentiation and proliferation through multiple pathways (Shi and Chen, 2014). For example, it has been shown that TGF-β control element (TCE), a smooth muscle (SM) alpha-actin promoter required for the TGF-β inducibility of SM alpha-actin in SM cells, is needed for gene transcription in VSMC (Liu et al., 2003). KLF4 is a trans-acting factor with TCE (Adam et al., 2000). Specificity protein (Sp) 1, a ubiquitous transcription factor that mediates the transcription of ECM genes (Verrecchia et al., 2001), forms a complex with KLF4 and binds TCE at the promoter of the angiotensin II type 1 receptor (AT1R) in the absence of TGF-β to maintain a basal expression level of AT1R in VSMC. Upon TGF-β activation, the expression of AT1R is suppressed through TGF-β1-mediated dissociation of the KLF4-Sp1 complex from TCE at the AT1R promoter (Zhang et al., 2012). Moreover, in VSMC, phosphorylated KLF4 can activate the TGF-β1 receptor promoter by interacting with Smad2 (Zhang et al., 2012). Furthermore, KLF4 exhibited an inhibitory effect on the proliferation of VSMC via binding to p53 (Yoshida et al., 2008).

Serum response factor (SRF) regulates the expression of VSMC differentiation markers by cooperating with its co-activator, myocardin, or its co-repressor, phosphorylated KLF4 (Wang et al., 2001). KLF4 also suppresses the expression of VSMC contractile markers by interacting with SRF (Hu et al., 2014). Therefore, KLF4 plays a catalytic role in renal fibrosis by inhibiting the differentiation and proliferation of VSMC.

### KLF4 in renal fibrosis

Recently, Chen et al. showed that KLF4 was indeed involved in the regulation of **renal** physiological functions and the progression of fibrosis (Chen et al., 2015). In two *in vivo* models of unilateral ureteral obstruction, a decrease in KLF4 expression was observed (Chen et al., 2015) and (Xiao et al., 2015), indicating that KLF4 has anti-fibrotic action in the kidney. Nevertheless, the role of KLF4 in renal fibrosis is still not clear, there is a need for further study.

## Conclusions and perspectives

The role of KLF4 in renal fibrosis is complex. For instance, KLF4 can induce the transformation of primary fibroblasts (Rowland et al., 2005) but can also induce fibroblasts to become pluripotent stem cells in mice (Takahashi and Yamanaka, 2006; Okita et al., 2007; Wernig et al., 2007). These findings make the function of KLF4 in renal fibrosis confusing; nevertheless, the findings described above indicate that KLF4 participates in the development of renal fibrosis and that its inhibition of fibrosis is greater than its promotion of the condition.

Because KLF4 is expressed in kidney cells (Hayashi et al., 2014), the ability to reprogramming somatic cells into IPS cells makes it possible to utilize KLF4 as a novel therapeutic target for Chronic kidney disease (CKD; D'Anselmi et al., 2013; Yamaguchi et al., 2014). Moreover, epigenetic modifications are known to play an essential role in kidney function and development (Dressler, 2008), and overexpression of KLF4 can reduce DNA methylation at the nephrin promoter, increasing the expression of nephrin and reducing the expression of mesenchymal genes (Hayashi et al., 2014). KLF4 and epigenetic modulation can thus be targeted as part of an intervention to treat proteinuria (Hayashi and Itoh, 2015). In addition, KLF4 can reduce the inflammation stimulated by TGF-β1 in cases of renal fibrosis caused by diabetic nephropathy (Mreich et al., 2015). Taken together, these findings strongly suggest that KLF4 represents a potential therapeutic target for renal fibrosis.

### Conflict of interest statement

The authors declare that the research was conducted in the absence of any commercial or financial relationships that could be construed as a potential conflict of interest.
