# Corrigendum: The Role of Krüppel-like Factor 4 in Renal Fibrosis

**DOI:** 10.3389/fphys.2016.00059

**Published:** 2016-02-18

**Authors:** Ben Ke, Afei Zhang, Xianfeng Wu, Xiangdong Fang

**Affiliations:** Department of Nephrology, Second Affiliated Hospital to Nanchang UniversityNanchang, China

**Keywords:** KLF4, renal fibrosis, Inflammation, TGF-β, vascular disease

In this paper titled “The Role of Krüppel-like Factor 4 in Renal Fibrosis,” there was a mistake in the department, which should be corrected. The correct work units should be Department of Nephrology, Second Affiliated Hospital to Nanchang University, Nanchang, China. No other correction is needed as the text and figure legends are correct.

## Author contributions

All authors listed, have made substantial, direct and intellectual contribution to the work, and approved it for publication.

## Funding

This work was supported by grants from the National Natural Science Foundation of China (General Program 81460142).

### Conflict of interest statement

The authors declare that the research was conducted in the absence of any commercial or financial relationships that could be construed as a potential conflict of interest.

